# Supplementary material for: Investigating the Antibacterial Properties of Prospective Scabicides
Source: Biomedicines. 2022 Dec 19;10(12):3287. doi: 10.3390/biomedicines10123287 (PMC9776028; doi:10.3390/biomedicines10123287)
Supplement: Supplementary file 1 [file biomedicines-10-03287-s001.zip › biomedicines-2056077-supplementary.pdf]

Supplementary Materials

Investigating the Antibacterial Properties of Prospective Scabicides

Sara Taylor, Deonne Walther, Kylie Agnew-Francis, Deepani D. Fernando, Pearl Swe-Kay, Sharon Chow, Craig Williams and Katja Fischer

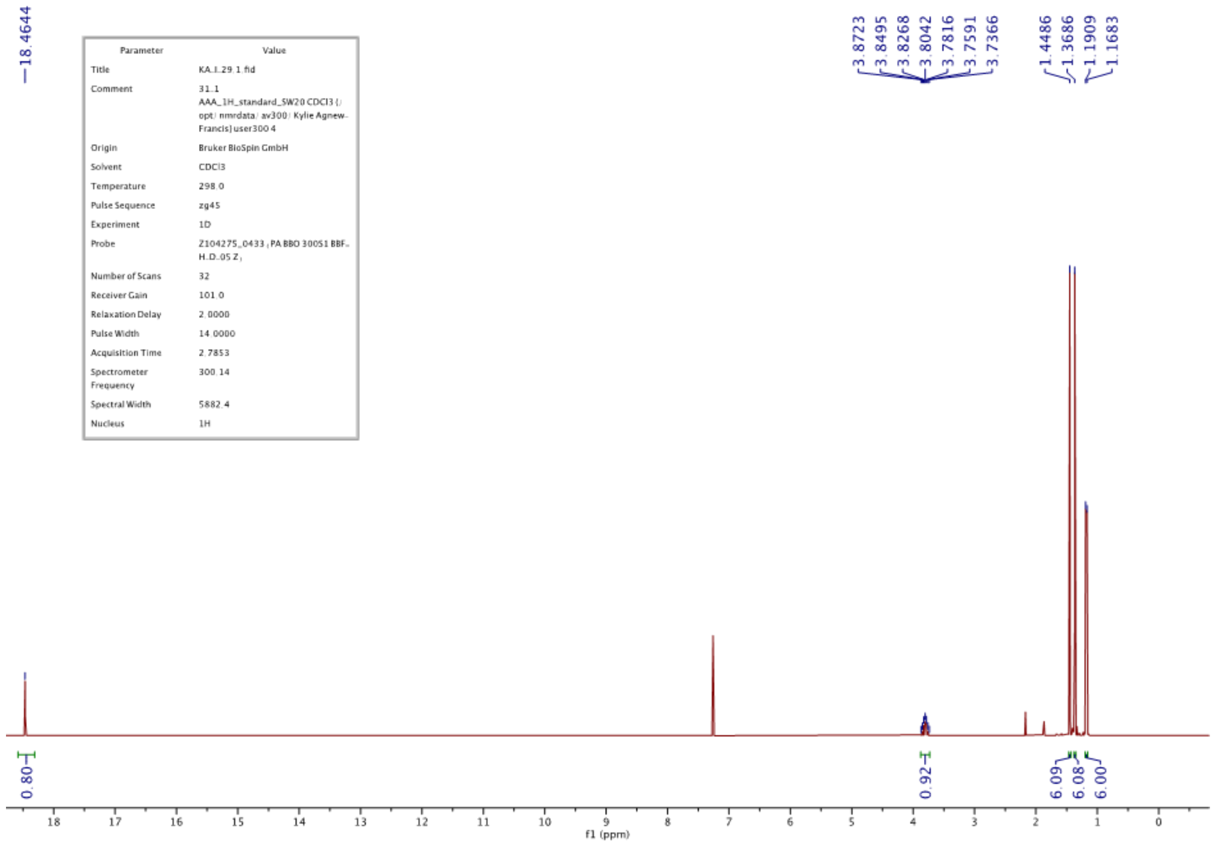

Figure S1. ^{1}H NMR of Flavesone.

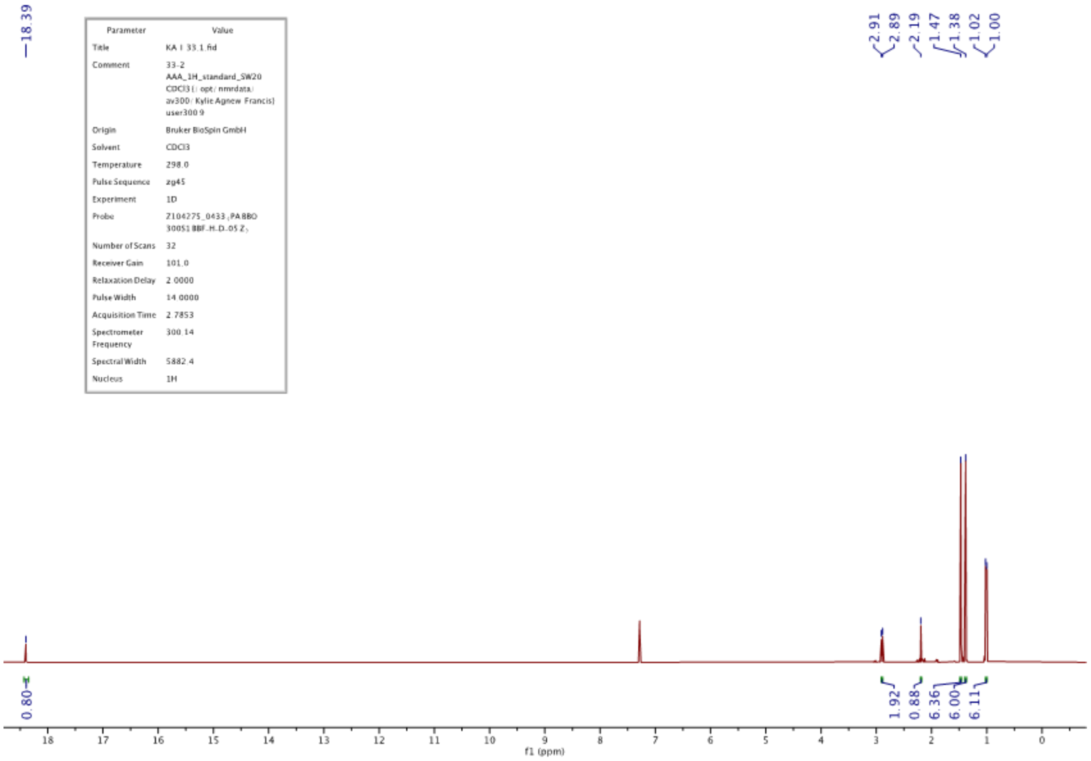

Figure S2. ^{1}H NMR of Leptospermone.

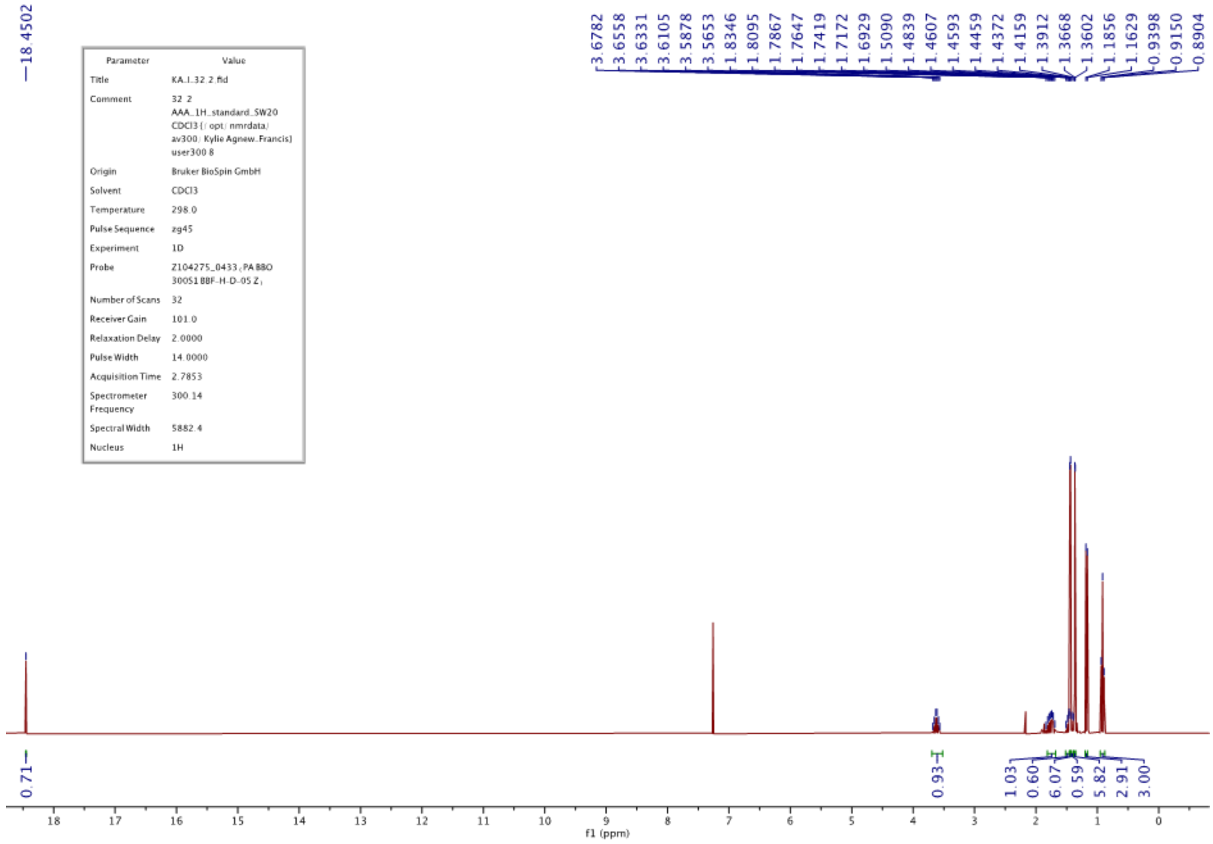

Figure S3. ^{1}H NMR of Isoleptospermone.

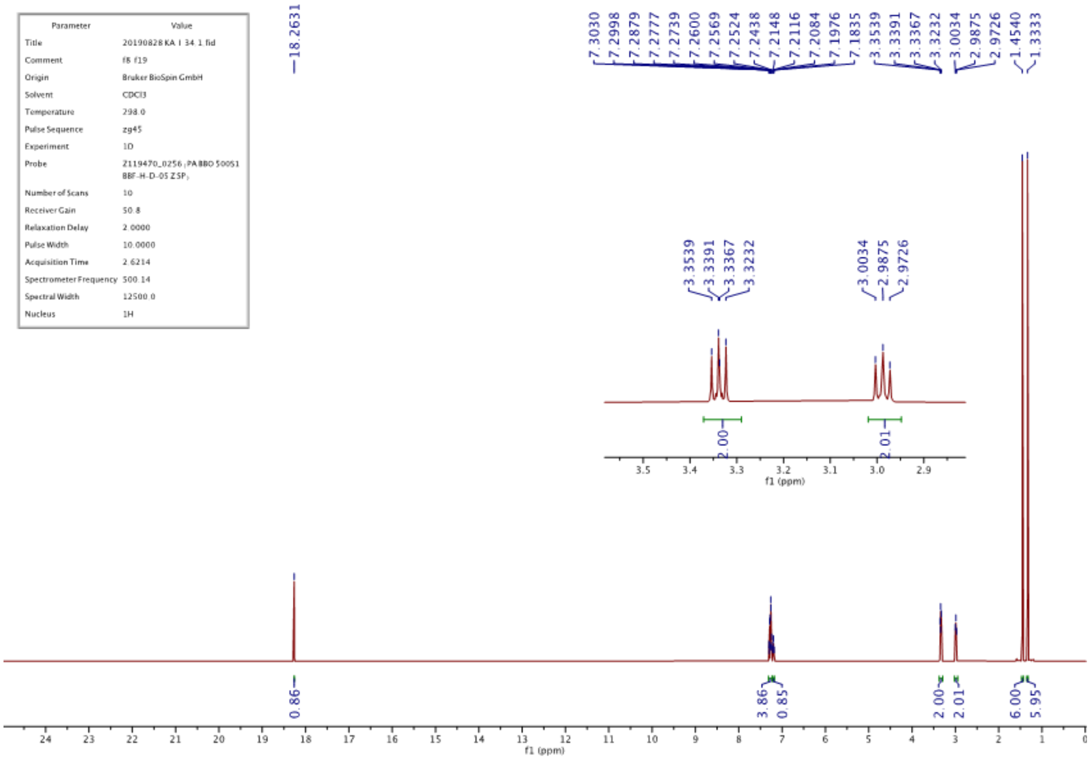

Figure S4. ^{1}H NMR of Grandiflorone.

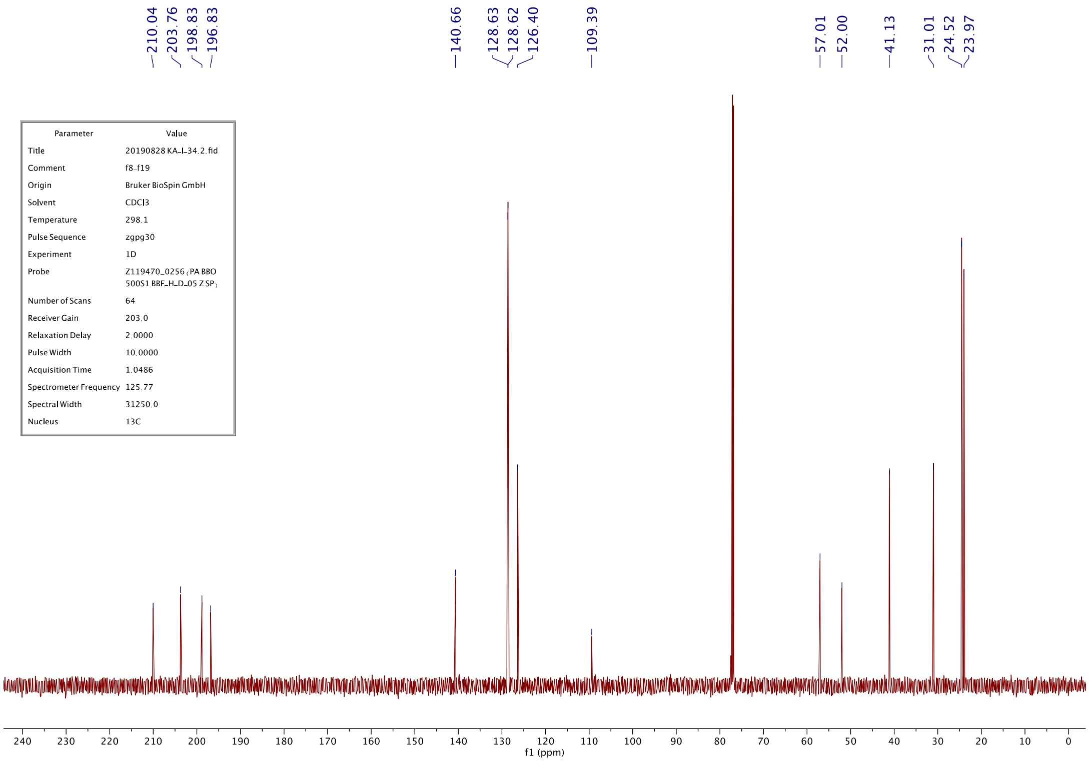

Figure S5. ^{13}C NMR of Grandiflorone.

Table S1. Vehicle controls and antibiotic controls.

	A.baumannii* *ATCC19606		***A.baumannii* *ATCC17978***		***A.baumannii* *BAA1605***		***S. aureus* XEN29 NCTC8532**		***S. aureus* MSSA CC75 M5**		***S. aureus* MRSA CC75 M34**		***S. pyogenes* *2031***		***S. pyogenes* *2967***		***S. pyogenes* *8830***		***S. dysgalactiae* *MD10***		***S. dysgalactiae* *ns3396***	
	MIC (% v/v)	MBC (% v/v)	MIC (% v/v)	MBC (% v/v)	MIC (% v/v)	MBC (% v/v)	MIC (% v/v)	MBC (% v/v)	MIC (% v/v)	MBC (% v/v)	MIC (% v/v)	MBC (% v/v)	MIC (% v/v)	MBC (% v/v)	MIC (% v/v)	MBC (% v/v)	MIC (% v/v)	MBC (% v/v)	MIC (% v/v)	MBC (% v/v)	MIC (% v/v)	MBC (% v/v)
DMSO	12.5 ±0	25 ±0	12.5 ±0	25 ±0	12.5 ±0	25 ±0	12.5 ±0	25 ±0	12.5 ±0	25 ±0	12.5 ±0	25 ±0	12.5 ±0	25 ±0	12.5 ±0	25 ±0	12.5 ±0	25 ±0	12.5 ±0	25 ±0	12.5 ±0	25 ±0
Isopropanol	6.25 ±0	12.5 ±0	6.25 ±0	12.5 ±0	6.25 ±0	12.5 ±0	12.5 ±0	25 ±0	12.5 ±0	25 ±0	12.5 ±0	25 ±0	n/a	n/a	n/a	n/a	n/a	n/a	n/a	n/a	n/a	n/a
	MIC (µg/ml)	MBC (µg/ml)	MIC (µg/ml)	MBC (µg/ml)	MIC (µg/ml)	MBC (µg/ml)	MIC (µg/ml)	MBC (µg/ml)	MIC (µg/ml)	MBC (µg/ml)	MIC (µg/ml)	MBC (µg/ml)	MIC (µg/ml)	MBC (µg/ml)	MIC (µg/ml)	MBC (µg/ml)	MIC (µg/ml)	MBC (µg/ml)	MIC (µg/ml)	MBC (µg/ml)	MIC (µg/ml)	MBC (µg/ml)
Ampicillin	n/a	n/a	n/a	n/a	n/a	n/a	1.2	1.2	1.2	1.2	13.3 ±5	32 ±9.6	1.2	1.2	1.2	1.2	1.2	2.4 ±0	1.2	1.2	1.2	1.2
Ampicillin + Sulbactam	12	19.2 ±0	4 ±1	14.4 ±5.3	39	153.6 ±0	n/a	n/a	n/a	n/a	n/a	n/a	n/a	n/a	n/a	n/a	n/a	n/a	n/a	n/a	n/a	n/a
